# Visualization of lymphatic flow in laparoscopic colon cancer surgery using indocyanine green fluorescence imaging

**DOI:** 10.1038/s41598-020-71215-3

**Published:** 2020-08-31

**Authors:** Hokuto Ushijima, Junichiro Kawamura, Kazuki Ueda, Yoshinori Yane, Yasumasa Yoshioka, Koji Daito, Tadao Tokoro, Jin-ichi Hida, Kiyotaka Okuno

**Affiliations:** Department of Surgery, Kindai University Faculty of Medicine, 377-2 Ohno higashi, Osakasayama, Osaka 589-8511 Japan

**Keywords:** Cancer, Surgical oncology, Materials science

## Abstract

Intraoperative visualization of lymphatic flow could guide surgeons performing laparoscopic colon cancer surgery on the extent of intestinal resection required. The purpose of this study was to investigate indocyanine green fluorescence imaging for intraoperative detection of lymphatic flow and nodes in such patients. All patients undergoing elective laparoscopic surgery for colorectal cancer from October 2016 to July 2017 were included in this study. Indocyanine green was injected submucosally around the tumors via a colonoscope and lymphatic flow assessed with a laparoscopic near-infrared camera system intraoperatively. Lymphatic flow was visualized perioperatively in 43 of 57 patients (75.4%). The rate of visualized lymphatic flow was significantly higher in patients with a lower clinical stage than in those with a higher clinical stage (*p* = 0.0103). Among the 14 patients in whom lymphatic flow was not visualized, 10 (71.4%) had cStage III or IV cancer. Our results indicate the potential role of intraoperative navigation in colon cancer surgery in early-stage colon cancers. This method allows the surgeon to clearly identify lymphatic flow during surgery and allows the determination and individualization of the lymph node dissection range.

## Introduction

The morbidity and mortality rates of colorectal cancer (CRC) are increasing worldwide^1^. The incidence and mortality rate of CRC are likewise rising in Japan^2^. In Europe, complete mesocolic excision (CME) in conjunction with central vascular ligation was first described a decade ago, and true central ligation of the main arteries and veins at their root was emphasized with sharp dissection along the mesocolic plane^3,4^. In Japan and many Asian countries, D3 lymphadenectomy has been the standard procedure for advanced CRC^5^. Japanese D3 surgery is based on principles similar to those of CME with central vascular ligation, and impressive outcomes have been reported^6^. D3 Lymphadenectomy includes pericolic, intermediate, and main node dissection, and the range of ligation of vessels are different in patients with CRC depending on the tumor location. When the tumor is located in the hepatic flexure, lymphatic flow may be found in the accessory right colic vein, right branch of the middle colic artery, and right colic artery. In addition to the regions along these vessels, extramesocolic lymph node metastasis can be found in the region along the right gastroepiploic artery in some patients^7^.

The lymphatic flow, along which lymph node metastasis of carcinomas can occur, varies among individual patients and even among tumors located at the same site. However, a method of intraoperative lymphatic flow diagnosis for patients with CRC has not been established. The use of near-infrared (NIR) fluorescence after the injection of indocyanine green (ICG) was recently reported for intraoperative sentinel lymph node mapping in patients with colon cancer^8^. A few studies have demonstrated the visualization of lymphatic flow in patients with CRC using ICG fluorescence imaging (ICG-FI)^9,10^. Intraoperative visualization of lymphatic flow could help surgeons to accurately identify individual patients’ lymphatic flow and change the extent of intestinal resection.

The purpose of this study was to investigate the intraoperative detection of lymphatic flow and lymph nodes using ICG-FI in patients with colon cancer.

## Materials and methods

### Patients

Patients were eligible for enrollment if they were ≥ 20 years old, had histologically confirmed CRC, had adequate critical organ functions, and had an Eastern Cooperative Oncology Group performance status of 0 or 1. Patients were excluded if written informed consent could not be obtained from them, if they were allergic to ICG or iodine, or if they were pregnant or lactating. The study was carried out in accordance with the Declaration of Helsinki on experimentation with human subjects and was approved by the Institutional Ethics Review Board of Kindai University (Approval No. 28-109). It was registered at the UMIN Clinical Trials Registry as UMIN 000025300 (https://www.umin.ac.jp/ctr/index.htm). Written informed consent was obtained from all patients at the time of enrollment.

All eligible patients who were diagnosed with CRC and underwent elective laparoscopic surgery at Kindai University Hospital from October 2016 to July 2017 were enrolled in this study. The cancer location was diagnosed by colonoscopy, and the clinical stage was diagnosed by enhanced computed tomography, positron emission tomography–computed tomography, and/or magnetic resonance imaging.

### Surgical procedures

The intestinal resection areas and lymph node dissection areas were determined in accordance with the tumor staging described in the Japanese Society for Cancer of the Colon and Rectum guidelines^5^. Lymph node dissection was performed regardless of the fluorescence results. When lymphatic flow by ICG-FI was observed beyond the D3 dissection range, lymph node dissection in the area along the lymphatic flow was added if clinically feasible. For example, if lymphatic flow to the right gastroepiploic artery region was observed in a patient with right-sided transverse colon cancer, lymph node dissection in the same region was added to the treatment protocol.

### ICG fluorescence imaging

**Figure 1:**
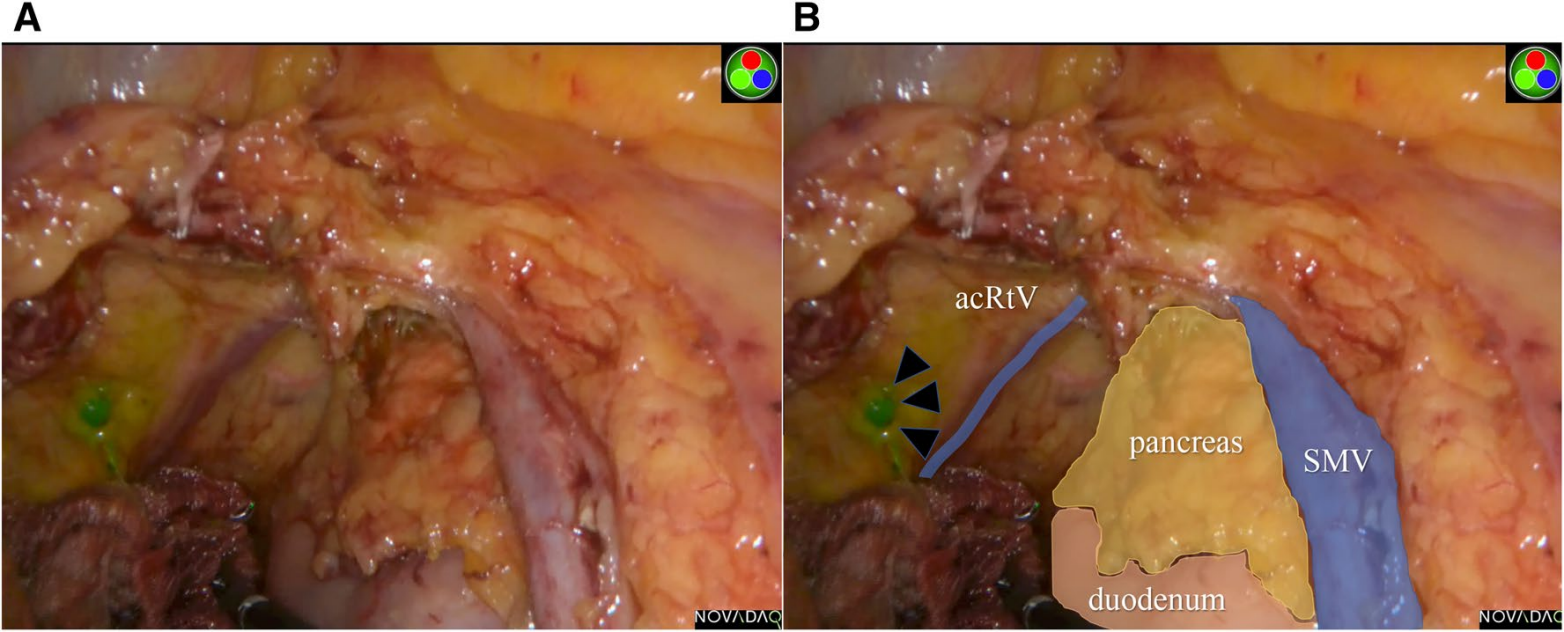
Intraoperative findings during right hemicolectomy. (**A**) The lymphatic flow and lymph node were visualized intraoperatively using the NIR camera system. (**B**) The relationship between the lymph node (arrows) and the surrounding organs and vessels were colored. *acRtV* accessory right vein, *SMV* superior mesenteric vein.

**Figure 2:**
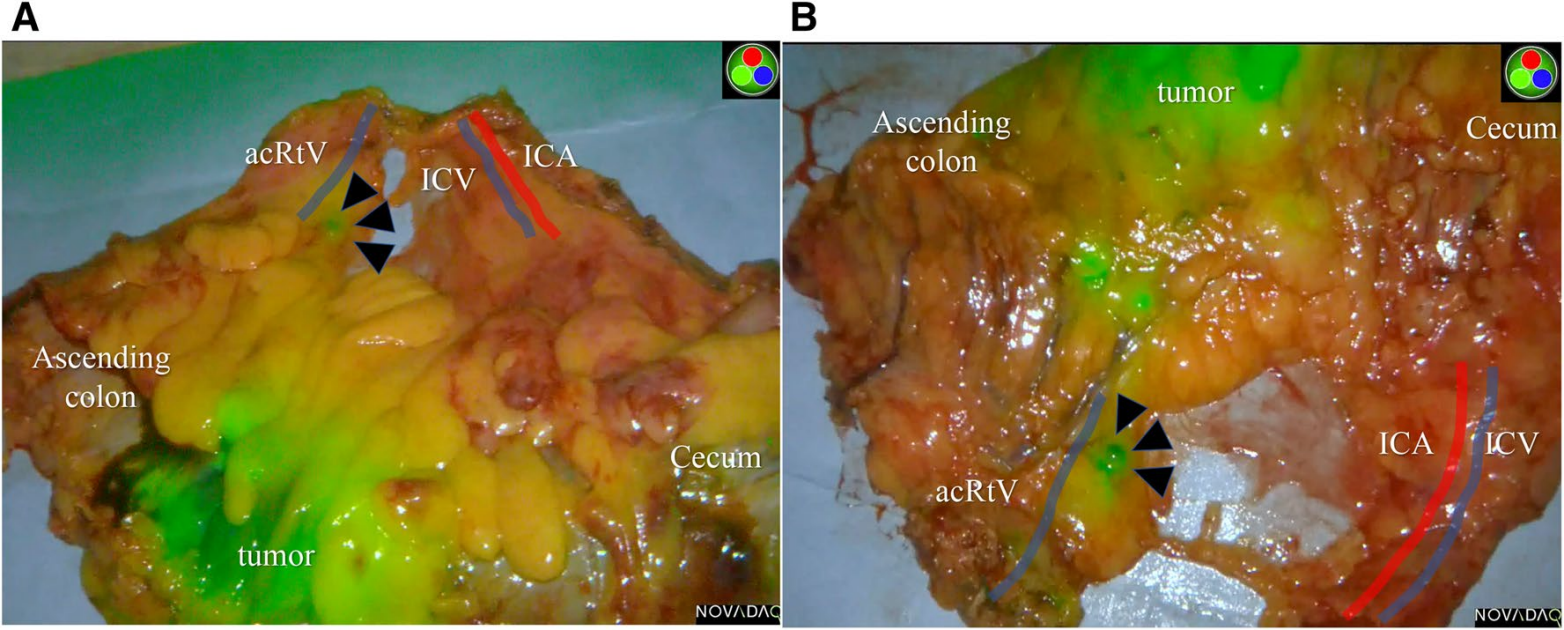
A retrieved specimen was scanned by the near-infrared camera system. The lymph node visualized during surgery was indicated by arrows, and feeding artery and drainage vein were colored. (**A**) Front side, (**B**) backside. *acRtV* accessory right vein, *ICA* ileocolic artery, *ICV* ileocolic vein.

For the detection of lymphatic flow, 0.2 to 0.3 mL of ICG solution (2.5 mg/mL) was injected into the submucosal layer just beneath the tumor at a single site preoperatively, using an injection needle under colonoscopy. ICG injection was performed 1 or 2 days before surgery. Lymphatic flow was confirmed using a PINPOINT laparoscopic NIR camera system (NOVADAQ, Ontario, Canada) (Fig. 1). After removal of the specimen, the lymph nodes were extracted and observed with the same NIR camera to confirm the existence of fluorescence (Fig. 2).

The fluorescence of lymphatic flow was confirmed by all surgeons [operator, assistant, and camera assistant including the first author (H.U.)] at the time of the first intra-abdominal observation. In terms of lymph nodes, the fluorescence of them was confirmed ex vivo after specimen removal in a nodule-by-nodule fashion.

### Statistical analysis

Data are presented as median and range. Qualitative data were reported as the number of patients (percentage of patients) and were compared with either the Pearson χ^2^ test or Fisher’s exact test, as deemed appropriate. Statistical analyses were performed using JMP 13 software (SAS Institute Inc., Cary, NC, USA).

## Results

In total, 57 patients with CRC were analyzed. The patients’ characteristics are shown in Table 1. Among the 57 patients, 33 (57.9%) were male and 24 (42.1%) were female. The median age was 71.5 years (range 45–94 years), and the median body mass index (BMI) was 22.9 kg/m^2^ (range 15.7–30.4 kg/m^2^). The tumor locations included the cecum in 5 (8.8%) patients, ascending colon in 16 (28.1%), transverse colon in 10 (17.5%), descending colon in 5 (8.8%), and sigmoid colon in 21 (36.8%) (Table 1). None of the patients underwent conversion to an open procedure. All procedures were safely performed without any complications. Preoperative chemotherapy was performed in two (3.5%) patients (modified FOLFOX6 plus bevacizumab, n = 1; XELOX, n = 1). In four patients, simultaneous laparoscopic hepatectomy was performed for metastatic tumors. No adverse reactions to the ICG injection were observed in any of the patients.

**Table 1:** Clinical characteristics and tumor locations.

Variables	N = 57
Age, years	71.5 (45–94)
**Gender**	
Male	33 (57.9)
Female	24 (42.1)
Body mass index, kg/m^2^	22.9 (15.7–32.6)
**Tumor location**	
Cecum	5 (8.8)
Ascending colon	16 (28.1)
Transverse colon	10 (17.5)
Descending colon	5 (8.8)
Sigmoid colon	21 (36.8)

Data are expressed as median (range) or n (%).

The lymphatic flow was observed at the time of the first intra-abdominal exploration. The lymphatic flow was visualized perioperatively in 43 (75.4%) patients and was not visualized in 14 (24.6%) patients. The relationship between the visualized lymphatic flow and the clinicopathological factors is shown in Table 2. The rate of visualized lymphatic flow was significantly higher in patients with a lower clinical stage than in those with a higher clinical stage (*p* = 0.0103). Among the 14 patients in whom lymphatic flow was not visualized, 10 (71.4%) had cStage III or IV cancer as shown in Table 3. There were no significant differences in the rate of visualized lymphatic flow between patients with high BMI (≥ 22.9 kg/m^2^) and low BMI (< 22.9 kg/m^2^), right- and left-sided tumors, and injection time in day 1 or day 2 before surgery (Table 2).

**Table 2:** Correlation between clinicopathological findings and lymphatic flow.

	Lymphatic flow		*P*-value
	Present	Absent	
**Tumor location**			
Right side (C, A, T)	26	5	
Left side (D, S)	17	9	0.1057
**cStage**			
0–II	29	4	
III, IV	14	10	0.0103^b^
**pStage**			
0–II	31	7	
III, IV	12	7	0.1348
**Body mass index**^a^			
High (≥ 22.9)	21	8	
Low (< 22.9)	22	6	0.4890
**Lymphatic invasion**			
Negative	27	5	
Positive	16	9	0.0763
**Injection timing**			
1 day before	32	8	
2 days before	11	6	0.2294
**CEA (ng/ml)**			
> 5	17	7	
≤ 5	26	7	0.4927

^a^BMI median 22.9, BMI high ≥ 22.9, BMI low < 22.9.

^b^A *P* value less than 0.05 was considered significant.

**Table 3:** Clinicopathologic characteristics of patients with non-visualized lymphatic flow.

No.	Lymphatic flow	cTNM/ cStage	pTNM/ pStage	Tumor location/ operation
1	–	T1b, N0, M0, H0/ I	T1b, N0, M0, H0/ I	A/ lap-Rt
2	–	T1b, N0, M0, H0/ I	T4a, N0, M0, H0/ II	A/ lap-Rt
3	–	T1b, N0, M0, H0/ I	T1b, N0, M0, H0/ I	S/ lap-S
4	–	T2, N0, M0, H0/ I	T2, N0, M0, H0/ I	S/ lap-HAR
5	–	T3, N1, M0, H0/ IIIa	T3, N0, M0, H0/ II	S/ lap-HAR
6	–	T3, N1, M0, H0/ IIIa	T3, N0, M0, H0/ II	S/ lap-S
7	–	T3, N0, M0, H0/ IIIa	T3, N1, M0, H0/ IIIa	S/ lap-S
8	–	T4a, N2, M0, H0/ IIIa	T3, N2, M0, H0/ IIIb	C/ lap-C
9	–	T3, N2, M0, H0/ IIIb	T1b, N0, M0, H0/ I	A/ lap-Rt
10	–	T4b, N2, M0, H0/ IIIb	T3, N1, M0, H0/ IIIa	A/ lap-Rt
11	–	T1a, N1. M1, H0/ IV	T1a, N0, M1, H1/ IV	S/ lap-S
12	–	T3, N1, M1, H0/ IV	T4a, N2, M1, H0/ IV	S/ lap-S
13	–	T4a, N2, M1, H1/ IV	T4a, N3, M1, H1/ IV	S/ lap-S
14	–	T4a, N2, M1, H0/ IV	T4a, N2, M0, H0/ IIIb	S/ lap-HAR

*C* cecal cancer, *A* ascending colon cancer, *T* transverse colon cancer, *D* descending colon cancer, *S* sigmoid colon cancer, *lap-C* laparoscopic ileocecal resection, *lap-Rt* laparoscopic right hemicolectomy, *lap-T* laparoscopic transverse colon resection, *lap-Lt* laparoscopic left hemicolectomy, *lap-S* laparoscopic sigmoid colon resection, *lap-HAR* laparoscopic high anterior resection.

Seventeen patients presented pathological lymph node metastasis (Table 4). Of these, lymphatic flow was visualized in 11 (64.7%) patients. All metastatic lymph nodes were identified in the fluorescent-marked lymphatic area. In the case of patient No. 6, who underwent right hemicolectomy for ascending colon cancer, the lymphatic flow was observed in the right branch of the middle colic artery, and lymph node metastasis was identified within the same area. This finding indicated that fluorescent lymphatic flow was consistent with lymph node metastasis. However, in patients No. 8 and No. 10 who presented left-sided transverse colon cancers at approximately at the same site and had the same pathological stage, the lymphatic flows differed. This phenomenon indicated that the lymphatic flow at the tumor may change in cases of metastatic lymph nodes.

**Table 4:** Clinicopathologic characteristics of patients with cancer cell positive lymph nodes.

No.	pTMN/ cStage	Lymphatic flow	Fluorescence region	LN metastasis	Tumor location/ operation
1	T2, N1, M0/ IIIa	+	ICA	Paracolic LNs	C/ lap-C
2	T3, N1, M0/ IIIa	+	ICA	Paracolic LNs	C/ lap-C
3	T3, N2, M0/ IIIa	+	acRtV	Central LNs of RCA	A/ lap-Rt
4	T3, N1, M0/ IIIa	+	acRtV	Paracolic LNs	A/ lap-Rt
5	T4a, N1, M0/ IIIa	+	RCA	Paracolic LNs	A/ lap-Rt
6	T3, N1, M0/ IIIa	+	MCA Rt	Intermediate LNs of MCA	A/ lap-Rt
7	T1b, N1, M0/ IIIa	+	MCA Rt	Paracolic LNs	T/ lap-T
8	T3, N1, M0/ IIIa	+	MCA Lt	Paracolic LNs	T/ lap-Lt
9	T4a, N1, M1/ IV	+	MCA Lt	Paracolic LNs	T/ lap-Lt
10	T3, N1, M0/ IIIa	+	LCA	Paracolic LNs	T/ lap-Lt
11	T4b, N1, M1/ IV	+	IMA	Paracolic LNs	S/ lap-S
12	T3, N2, M0/ IIIb	−	–	Paracolic LNs	C/ lap-C
13	T3, N1, M0/ IIIa	−	–	Paracolic LNs	A/ lap-Rt
14	T3, N1, M0/ IIIa	−	–	Paracolic LNs	S/ lap-S
15	T4a, N2, M1/ IV	−	–	Paracolic LNs, intermediate LNs of IMA	S/ lap-S
16	T4a, N3, M1/ IV	−	–	Paracolic LNs	S/ lap-S
17	T4a, N2, M1/ IV	−	–	Paracolic LNs	S/ lap-Hartmann

*C* cecal cancer, *A* ascending colon cancer, *T* transverse colon cancer, *D* descending colon cancer, *S* sigmoid colon cancer, *lap-C* laparoscopic ileocecal resection, *lap-Rt* laparoscopic right hemicolectomy, *lap-T* laparoscopic transverse colon resection, *lap-Lt* laparoscopic left hemicolectomy, *lap-S* laparoscopic sigmoid colon resection, *RCA* right colic artery, *MCA* middle colic artery, *IMA* inferior mesenteric artery.

The correlation between ICG fluorescent positivity and cancer cell positivity for lymph nodes is shown in Table 5. A total of 222 (15.6%) ICG fluorescent-positive nodes were identified in 1,425 nodes obtained from resected specimens irrespective of cancer cell positivity. The technique identified 12 ICG fluorescent/cancer cell-positive nodes (17.6%) among a total of 68 cancer cell-positive nodes, while there were 1,147 ICG non-fluorescent/cancer cell-negative nodes (84.5%) among a total of 1,357 cancer cell-negative nodes.

**Table 5:** Correlation between ICG fluorescence and cancer cell positivity in the lymph nodes.

	Cancer cell positivity		Total
	Positive	Negative	
**ICG**			
Fluorescent	12	210	222
Non-fluorescent	56	1,147	1,203
Total	68	1,357	1,425

*ICG* indocyanine green.

## Discussion

ICG fluorescence imaging has been used in innovative surgical techniques including perioperative blood flow assessment in coronary artery bypass grafting, intraoperative imaging during flap plasty, and detection of sentinel lymph nodes^11,12^. Additionally, ICG fluorescence imaging has been used to assess the blood flow of the anastomotic sites in patients with CRC^10^. A few studies have described the visualization of lymphatic flow by ICG fluorescence imaging in patients with CRC^9,10^. In this study, lymphatic flow was visualized in approximately 75% of all patients. The rate of visualized lymphatic flow was significantly higher in patients with lower clinical stage than in those with higher clinical stage. Patients with cStage III and IV had a higher incidence of non-visualized lymphatic flow. These results suggested that the ICG fluorescent imaging technique had insufficient detectability for lymph flow in patients at higher clinical stage. However, our results indicate a potential role for ICG for intraoperative navigation during colon cancer surgery in select patients with clinically node-negative colon cancers.

When lymph node metastasis occurs, the lymph node can become filled with cancer cells, and the lymphatic flow is likely to disappear. In fact, we reviewed the status of lymphatic invasion for all cases. Among the 43 patients with visualized lymphatic flow, 27 exhibited no lymphatic invasion (62.8%) and 16 patients presented with positive lymphatic invasion (37.2%) (Table 2), while of the 14 patients in whom lymphatic flow was not visualized, 5 presented with no lymphatic invasion (35.7%), while 9 (64.3%) exhibited positive lymphatic invasion. Therefore, whether the fluorescent lymphatic flow could be visualized or not was associated with the degree of microscopic lymphatic invasion. The same non-significant trend was observed among groups with or without pathological lymph node metastasis. These results indicated that the most suitable patients for visualization of lymphatic flow are likely those who have a clinically node-negative cancer.

Some studies have shown high sensitivity and specificity using ICG fluorescent imaging, although neither sensitivity nor specificity was markedly high in our study (Table 5). ICG fluorescent lymph nodes did not always indicate cancer-positive nodes and even ICG non-fluorescent lymph nodes could be cancer-positive nodes because ICG is not a cancer-specific fluorophore suitable for the visualization of lymphatic flow during laparoscopic surgery for colon cancer. Our main purpose was to detect the lymphatic flow using the technique to determine which cancer cells could potentially metastasize to the lymph nodes. An expected role for this technique might be the optimization of the extent of lymph node dissection based on the lymphatic flow observed around the tumor in an individual patient.

In a previous study, ICG was injected into the subserosa through the trocars used during the operation or into the submucosa through colonoscopy^9^. In the present study, we preferred to inject ICG using a colonoscope 1 or 2 days before surgery. We believe that ICG injection using colonoscopy enables accurate injection just beneath the tumor site and leads to more precise diagnosis of the lymphatic regions.

This study has some limitations. First, the number of patients was small, and the follow-up period was short. Second, whether lymphatic flow could be observed in other ICG fluorescence imaging devices should be verified in further studies. Third, some technical errors and biases could not be ignored, particularly, in terms of the insufficient detectability of lymph flow. There may also be potential biases in our study due to the failure of ICG injection, the presence of a localized thick layer of mesorectal fat, or insufficient observation of fluorescence.

## Conclusions

We visualized intraoperative lymphatic flow in patients undergoing CRC surgery. Our results indicate the potential role of intraoperative navigation using ICG in colon cancer surgery in early-stage colon cancers. This method allows the surgeon to clearly identify lymphatic flow during surgery and allows the determination and individualization of the lymph node dissection range. However, further research is needed for selected patients with CRC.
